# Adipose-Derived Stem Cell, Stromal Vascular Fraction, and Regenerative Cell Enrichment in Fat Grafting: A Systematic Review of Safety and Functional Outcomes

**DOI:** 10.7759/cureus.99599

**Published:** 2025-12-19

**Authors:** Alaa H Hakami, Mohammed S Akkur, Khalid A Mahasi, Mayyas K Younis, Abdulaziz F AlDafas, Lara H Al Massloom, Bushra S Albakri, Hatoon Y Alajlan, Hadeel A Alamri, Sarah S Alqahtani, Meshary D Alzahrani

**Affiliations:** 1 Surgery, Jazan University, Jazan, SAU; 2 Medicine, Jazan University, Jazan, SAU; 3 Medicine, Sulaiman Alrajhi University, Qassim, SAU; 4 Medicine, King Saud Bin Abdulaziz University for Health and Science, Riyadh, SAU; 5 Medicine, Imam Abdulrahman Bin Faisal University, Dammam, SAU; 6 Radiology, Wadi Al Dawaser Armed Forces Hospital, Riyadh, SAU; 7 Medicine, Alryan National College, Medina, SAU; 8 Medicine, King Khalid University, Abha, SAU; 9 Medicine, Tabuk University, Tabuk, SAU

**Keywords:** adipose-derived regenerative cells, adipose-derived stem cells, aesthetic surgery, cell-assisted lipotransfer, fat grafting, lymphedema, reconstructive surgery, regenerative medicine, stromal vascular fraction, volume retention

## Abstract

Adipose-derived stem cell (ADSC)-enhanced fat grafting, including enrichment with stromal vascular fraction (SVF) and adipose-derived regenerative cells (ADRCs), has been increasingly used to improve fat graft survival and regenerative outcomes in reconstructive and aesthetic plastic surgery, yet long-term safety, efficacy, and mechanistic understanding remain variably reported. This systematic review aimed to evaluate real-world clinical evidence on the long-term safety, volume retention, and functional outcomes of ADSC-, SVF-, and ADRC-enriched fat grafting across aesthetic and reconstructive indications. Following the Preferred Reporting Items for Systematic Reviews and Meta-Analyses (PRISMA) 2020 guidelines, comprehensive searches of PubMed, Scopus, Web of Science, and the Cochrane Library identified 3158 studies, of which 12 met the inclusion criteria, encompassing randomized controlled trials and prospective non-randomized clinical studies assessing ADSC-, SVF-, or ADRC-assisted fat grafting with reported outcomes related to graft survival, clinical function, aesthetic performance, or safety. Across studies, ADSC and SVF enrichment generally improved volume retention compared with conventional fat grafting, with several randomized trials demonstrating graft survival rates of 60-80% at three to six months, and ADSC-assisted grafts consistently enhanced dermal regeneration by improving collagen organization, skin texture, and elastic fiber architecture. In reconstructive applications, SVF therapy reduced digital ulcers and pain in patients with systemic sclerosis, while ADRC-assisted grafting alleviated symptoms of breast cancer-related lymphedema despite limited objective limb-volume reduction, and patient satisfaction was high across all enrichment techniques. Safety outcomes were favorable, with no increase in infection, cyst formation, fat necrosis, or oncologic recurrence across follow-up periods of up to four years. Overall, ADSC-, SVF-, and ADRC-enriched fat grafting appear to offer meaningful improvements in graft survival, skin quality, and functional recovery with a strong safety profile across diverse clinical indications, although methodological heterogeneity and limited long-term data support the need for standardized, regulated, and mechanistically informed clinical protocols, as well as larger, long-term randomized trials to optimize techniques and guide clinical use.

## Introduction and background

Autologous fat grafting has become an essential technique in reconstructive and aesthetic plastic surgery, offering a biocompatible and natural option for soft-tissue volume restoration. Despite its versatility in facial rejuvenation, breast surgery, and correction of contour deformities, conventional fat grafting remains limited by inconsistent and unpredictable long-term volume retention. Graft survival rates vary widely, often necessitating repeat procedures and contributing to variable patient satisfaction. As a result, improving graft durability and integration has become a major focus in the evolution of fat grafting techniques [1-3].

Adipose-derived stem cell (ADSC)-based strategies have emerged as promising biologic approaches to address these limitations. ADSCs, which are multipotent mesenchymal cells found in high concentration within the stromal vascular fraction (SVF) of adipose tissue, possess regenerative properties that support angiogenesis, immunomodulation, and extracellular matrix remodeling [4-6]. These capabilities form the basis of cell-assisted lipotransfer (CAL), in which autologous fat is enriched with freshly isolated SVF, purified adipose-derived regenerative cells (ADRCs), or ex vivo-expanded ADSCs before grafting [1,7-10]. Early clinical and preclinical studies suggest that ADSC-enriched grafts may enhance neovascularization, reduce adipocyte apoptosis, and improve long-term graft survival [1-10].

Clinical applications of ADSC-enhanced fat grafting now span both aesthetic and reconstructive domains. In aesthetic practice, enrichment techniques have been applied to facial contouring, age-related volume loss, and breast augmentation, with reports of improved volume stability and skin quality [4,5,9,10]. In reconstructive settings, cell-assisted grafts have shown promise for post-radiation fibrosis, breast cancer-related lymphedema, scleroderma-related hand deformities, and severe facial lipoatrophy [2,3,6,11]. Despite this growing use, the evidence base remains fragmented, and questions persist regarding long-term safety, oncologic risk, standardization of cell preparation, and the magnitude of clinical benefit [1-12].

Significant heterogeneity across studies further complicates the interpretation of outcomes. Variations in cell isolation methods, fat processing techniques, injection protocols, follow-up duration, volumetric assessment tools, and outcome measures limit comparability and hinder the development of evidence-based guidelines [1-12]. A clear synthesis of real-world clinical data across both aesthetic and reconstructive applications is lacking.

Given the expanding interest in biologically enhanced fat grafting, a comprehensive evaluation of current clinical evidence is needed. This systematic review aims to assess the long-term safety, volume retention, and functional outcomes of ADSC-, SVF-, and ADRC-enriched fat grafting while identifying methodological limitations and outlining key areas for future research in cell-assisted fat grafting [1-12].

## Review

Methodology

Literature Search Strategy

This systematic review followed the Preferred Reporting Items for Systematic Reviews and Meta-Analyses (PRISMA 2020) guidelines [13]. A comprehensive search was conducted in PubMed, Scopus, Web of Science, and the Cochrane Library from inception to October 2025 to identify clinical studies evaluating ADSC-enhanced fat grafting. Searches included studies involving SVF, ADRCs, ex vivo-expanded ADSCs, and CAL in both aesthetic and reconstructive plastic surgery.

Search terms combined controlled vocabulary and free-text keywords related to ADSCs, SVF, ADRCs, fat grafting, and clinical outcomes such as graft survival and volume retention. Searches were limited to human studies published in English. Reference lists of included articles and relevant reviews were also screened to capture additional eligible studies.

Eligibility Criteria

Eligibility criteria were formulated using the PICO framework [14]. Included studies involved human participants undergoing autologous fat grafting for aesthetic or reconstructive indications, with interventions using ADSC-enhanced grafts through SVF, ADRCs, expanded ADSCs, or CAL techniques. Comparator groups could include standard fat grafting, placebo-enriched grafts, platelet-rich plasma (PRP), or medical therapy alone.

Eligible outcomes included volumetric assessment using MRI, CT, or validated 3D imaging, as well as clinical, aesthetic, functional, patient-reported, and safety outcomes. Prospective study designs, including randomized trials, nonrandomized comparative studies, and prospective feasibility studies, were included. Exclusion criteria comprised animal studies, in vitro research, reviews, conference abstracts, case reports, technique descriptions, and studies lacking measurable clinical outcomes or ADSC/SVF enrichment.

Study Selection

Two reviewers independently screened all identified records in a two-step process. Titles and abstracts were first assessed to remove irrelevant articles; remaining studies underwent full-text review to determine final eligibility. Disagreements were resolved through discussion or consultation with a third reviewer.

Data Extraction and Quality Appraisal

Data were extracted independently by two reviewers using a standardized form capturing study design, sample characteristics, clinical indication, type of ADSC or SVF enrichment, fat processing methods, injection technique, comparator details, volumetric outcomes, secondary outcomes, and follow-up duration. Discrepancies were resolved by consensus.

Risk of bias was evaluated using tools appropriate for each study design. Randomized trials were assessed using the Cochrane RoB 2 tool [15], and nonrandomized studies were evaluated with Risk Of Bias In Non-Randomized Studies - of Interventions (ROBINS-I) [16]. Each study was categorized as having low, moderate, or serious risk of bias, and only those meeting minimum quality thresholds were included in the final synthesis.

Results

Study Selection

**Figure 1:**
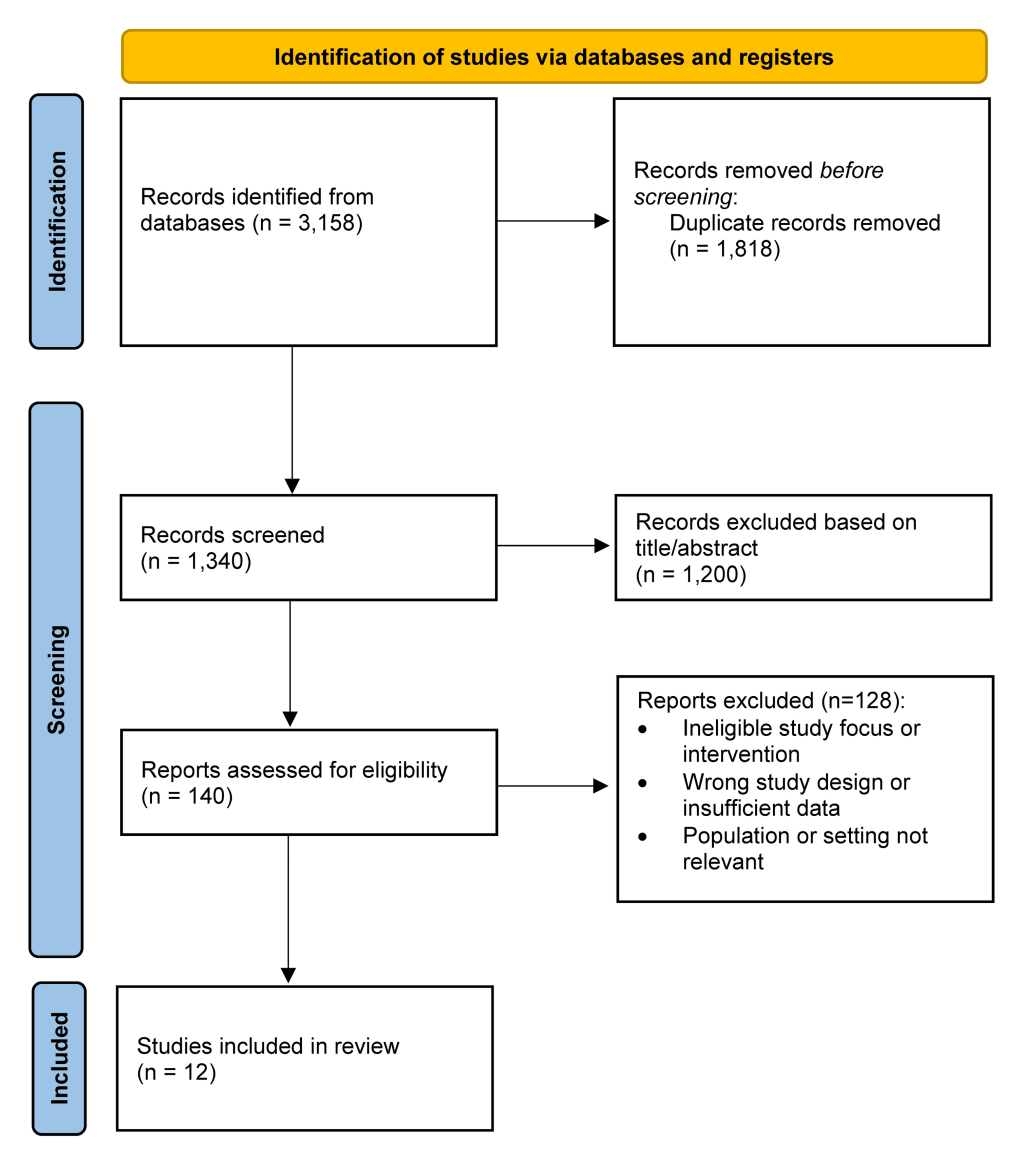
PRISMA flow diagram of the study selection process The PRISMA flowchart depicts the study selection process used in this review [13], outlining records identified through database searching and other sources, removal of duplicates, screening of titles and abstracts, full-text eligibility assessment, and final inclusion of studies, with reasons for exclusion reported at each step. PRISMA - Preferred Reporting Items for Systematic Reviews and Meta-Analyses

A total of 3158 records were identified across all databases. After removing duplicates, 1340 unique records were screened, and 1200 were excluded based on title and abstract. Full-text review of 140 articles resulted in the exclusion of 128 studies for reasons such as absence of ADSC or SVF enrichment, lack of clinical outcomes, use of in vitro or animal models, or publication as reviews or technique papers. Twelve studies [1-12] met all criteria for inclusion in the qualitative synthesis. No meta-analysis was performed due to substantial heterogeneity in study design, populations, enrichment methods, and outcome measures (Figure 1).

Baseline Characteristics

The included studies comprised randomized controlled trials, pilot feasibility studies, and prospective clinical series conducted across multiple continents. Sample sizes ranged from small split-body trials with approximately 10 participants to larger aesthetic and reconstructive trials enrolling up to 50 patients. Indications included facial augmentation, breast augmentation, facial lipoatrophy, systemic sclerosis, localized scleroderma, and breast cancer-related lymphedema. Most participants were young to middle-aged adults, predominantly women (Table 1).

**Table 1:** Summary of characteristics of the included studies This table summarizes clinical studies investigating ADSC- or SVF-enriched autologous fat grafting for various indications, including cosmetic augmentation, facial rejuvenation, lymphedema, and scleroderma-related atrophy. Study characteristics include country/setting, design, sample size and demographics (N = number of participants; age in years; sex; BMI in kg/m²), patient population, intervention type, comparator, technical details of harvesting, processing, and injection, primary outcomes (typically graft volume retention, expressed as %), secondary outcomes (clinical, functional, or aesthetic measures), follow-up duration, safety outcomes (adverse events (AEs), complications), and authors' conclusions. ADSC - adipose-derived stem cell; ADRC - adipose-derived regenerative cells; AFG - autologous fat grafting; CAL - cell-assisted lipotransfer; SVF - stromal vascular fraction; PRP - platelet-rich plasma; RCT - randomized controlled trial; LoS - localized scleroderma; BCRL - breast cancer-related lymphedema; MRI - magnetic resonance imaging; GA - general anesthesia; DASH - Disabilities of the Arm, Shoulder, and Hand score; GMP - good manufacturing practice; F - female; M - male; FNAC - fine-needle aspiration; ASC - adipose-derived stromal cells

Study ID (author)	Country / setting	Study design	Sample size & demographics (N, age, aex, BMI)	Patient population / indication	Intervention (type of ADSC enrichment)	Comparator	Technique details (harvesting, processing, injection)	Primary outcomes (volume retention %)	Secondary outcomes (clinical / functional / aesthetic)	Follow-up duration	Safety outcomes (complications, AEs)	Authors' conclusions
Wang et al. [1]	China - Peking Union Medical College Hospital (Beijing)	Prospective, randomized, controlled pilot trial (3-arm)	N=18 (6 conventional, 6 SVF-assisted, 6 ADSC-assisted) Age: 18–40 yrs, mean 26.7 ± 1.3 yrs Sex: 11 female, 7 male BMI: 21.22 ± 0.34 kg/m²	Localized scleroderma (LoS) patients with facial atrophy	ADSC-assisted AFG: Ex vivo expanded ADSCs (5 × 10⁵ cells/mL fat), P2-P3, viability >90%	1) Conventional AFG 2) SVF-assisted AFG (SVF: fat = 1:1)	Harvesting: low-pressure syringe liposuction from the abdomen. SVF isolation: collagenase digestion, viability 85–95%, markers: CD13+, CD29+, CD44+, CD73+, CD90+; CD34+ (>20%). ADSC expansion: serum-free medium, P2–P3; injection: 1.4-mm cannula, fan-shaped subcutaneous deposition, target graft volume +20%	6-month MRI: ADSC-assisted 49.83% ± 3.61%, SVF-assisted 31.75% ± 1.73% (p=0.0004 vs ADSC), conventional 21.86% ± 1.68% (p < .00001 vs ads)	Expert satisfaction is higher in the ADSC group; improved contour, symmetry, and fullness; MRI confirms a persistent adipose layer at 6 months	6 months	No infections, seromas, hematomas, or oil cysts; no serious adverse events	ADSC-assisted AFG is safe, feasible, and superior to SVF or conventional AFG for LoS facial atrophy; suggests a strong benefit of ADSC enrichment
Iglesias et al. [2]	Mexico - Instituto Nacional de Ciencias Médicas y Nutrición Salvador Zubirán	Open-label, randomized, controlled pilot clinical trial	N=20 (10 experimental, 10 control; 1 withdrawal → N=19). Age: ~55-57 yrs. Sex: 100% female (exp), 90% female (control). BMI: >18 kg/m²	Systemic sclerosis with hand involvement (pain, Raynaud's, ulcers, fibrosis, reduced mobility)	SVF-enriched microfat grafting: ADSVF isolated enzymatically + mixed with 40 cc fat	Control = medical therapy only	Harvesting: 100 mL of fat via syringe liposuction. Processing: Collagenase digestion → centrifugation → filtration → ADSVF isolation. Viable nucleated cells: 167.5×10⁶ (59-95% viability). Injection: 40 cc microfat + 2 mL ADSVF injected along neurovascular bundles	Pain: Significant reduction in the experimental group vs the control (p<0.05). Digital ulcers: Significant decrease at day 168 (p<0.01)	QoL (SF-36) improvement; Raynaud’s improved; skin score improved only in experimental; treated hands fuller, warmer, improved capillary refill	168 days (6 months)	No major complications. Donor site: mild pain & ecchymosis only (resolved 4-5 days). No serious vascular events	ADSVF + microfat grafting is safe and feasible. Improves pain and digital ulcers; larger trials needed for hand function outcomes.
Jørgensen et al. [3]	Denmark - Odense University Hospital	Prospective open-label phase I clinical trial (single arm)	N=10 Age median 55 yrs (range 34-68) Sex 100% female BMI median baseline 30.95 (range 22.32-35.91)	Breast cancer–related lymphedema (BCRL), unilateral, ISL stage I-II	ADRC-enriched lipotransfer (autologous ADRCs via Celution 800/CRS) + axillary scar-release lipotransfer	No control group	Harvesting: 280 g median lipoaspirate; water-jet assisted; no local anesthetic. Scar release: 30 mL of fat injected subcutaneously in the axilla. ADRC isolation: ~252 g processed; yield ≈5.32×10⁷ cells (~2.2×10⁵ cells/g). Injection: 4 mL ADRCs into 8 axillary points. Flow cytometry: CD34+, CD73+, CD90+, CD31-, CD45-	No significant reduction in lymphedema arm volume at 4 years	Improvements: arm heaviness ↓ 5.5→3.5, tension ↓ 5→2, DASH ↓ 21.25→9.17, 6/10 reduced compression therapy, cellulitis incidence ↓ 0.92→0.46/year (p=0.065), 5/10 felt significant improvement	1, 3, 6, 12 months & 4-year follow-up	No serious AEs. Minor liposuction discomfort; no locoregional cancer recurrence; one contralateral new primary, one distant recurrence (expected background risk)	ADRC-enriched lipotransfer is safe and feasible for BCRL, with sustained symptomatic improvement; no objective volume reduction. RCTs needed
Yin et al. [4]	China - Affiliated Hospital of Xuzhou Medical University	RCT (single-center, prospective, parallel)	N=50, all female; Age 20–55 yrs (SVF 35.5 ± 8.2; Control 35.3 ± 8.1); BMI 18.5–24 kg/m² (SVF 21.2 ± 1.8; Control 21.6 ± 1.9)	Facial rejuvenation requiring cosmetic fat grafting	Stromal vascular fraction (SVF)-enriched fat graft (fresh SVF, viability >98%)	Standard fat graft (no SVF)	Fat harvested from the lower abdomen (Coleman method). Experimental: 200-240 mL harvested; Control: 50-60 mL. SVF isolated in a GMP-grade lab and mixed with fat within 1.5 h. Injection volumes: Forehead 8-12 mL, Temporal 5-10 mL, Cheek 6-10 mL, Nasolabial 3-6 mL, Zygomatic 2-4 mL	Volume retention: SVF: 77.6% ± 11.6% vs Control: 56.2% ± 9.5% (p<0.001)	Enhanced improvement in wrinkles and texture (p<0.001 and p=0.003); both groups improved in spots, pores, UV spots, brown spots, and red areas. High patient satisfaction	6 months	No adverse events reported	SVF-assisted fat grafting significantly improves graft survival and skin quality; clinically effective and safe for facial rejuvenation.
Yoshimura et al. [5]	Japan - University of Tokyo + Kyorin University + Cellport Clinic Yokohama	Prospective clinical case series	N=40 healthy women,, Age: 20-62 yrs (mean 35.8 ± 9.1),, Sex: 40 female,, BMI: 19.7 ± 1.9	Patients undergoing cosmetic breast augmentation (not reconstruction)	SVF-enriched CAL (cell-assisted lpotransfer) using freshly isolated SVF mixed with half of the aspirated fat	None	Fat harvested via 2.5-mm cannula under GA; ~1,111 mL aspirated. Half used for SVF isolation (90 min processing). Fat was prepared either by washing or centrifugation, depending on the patient group: • Group A (n=6): washed fat + SVF • Group B (n=2): SVF injected diffusely after conventional lipoinjection • Group C (n=32): centrifuged fat + SVF pellets. Injection with an 18-G 150-mm needle in multiple planes. Mean injected volume: Left 268 mL; Right 277 mL.	Breast volume augmentation of 100-200 mL, stable after 2 months	High patient satisfaction; soft and natural contour; better augmentation than conventional lipoinjection (historical comparison). Improved adipose thickness on MRI and CT	Up to 42 months (19 patients ≥6 months)	Cyst formation <12 mm in 2 patients; microcalcifications in 2 patients at 24 months; 1 patient with fibrosis and stiffness in Group B. No major complications	CAL is effective and safe for soft tissue augmentation, with higher and stable breast volume. Centrifuged fat + SVF most effective
Yoshimura et al. [6]	Japan - University of Tokyo & affiliated hospitals	Prospective comparative clinical study (non-randomized; CAL vs non-CAL)	N=6 total; CAL group: n=3 (2 F, 1 M) age 33–48 (mean 38.7); Non-CAL group: n=3 (2 F, 1 M) age 25–55 (mean 46.3)	Severe acquired facial lipoatrophy due to lupus profundus (n=5) or Parry-Romberg syndrome (n=1)	SVF (ADSC-rich)–enriched fat grafting (CAL) using fresh SVF from half of the lipoaspirate	Conventional fat grafting (no SVF)	Harvesting: 2.5-mm cannula, saline + adrenaline under GA. SVF isolation: collagenase + centrifugation (800g × 10 min). Fresh SVF mixed with remaining fat, 10-15 min adherence. Fat centrifuged 700g × 3 min, kept cool. Injection: screw-type syringe, 18G needle, multilayer subcutaneous + muscle; ~20% overcorrection. Volume: Non-CAL 100–250 mL; CAL 90–110 mL	Clinical improvement score: CAL superior to non-CAL, p=0.11; all patients improved	Photo-based volumetric improvement: CAL Good–Excellent, non-CAL Fair–Good. Softness and natural texture are maintained	9-13 months	One non-CAL case fat necrosis requiring drainage; no infections or nodules in the CAL group	CAL is safe, enhances clinical outcomes, and appears superior to conventional fat grafting. Larger trials needed
Kølle et al. [7]	Denmark - Copenhagen University Hospital (Rigshospitalet)	Triple-blind, randomized, placebo-controlled trial	N=10 completed (13 enrolled). Age: mean 28.4 yrs (95% CI 22.0–34.8). Sex: 11 women, 2 men. BMI: mean 24.7 (95% CI 23.3–26.1)	Healthy volunteers receiving paired fat grafts in both upper arms for a controlled comparison of survival	Ex vivo expanded ADSCs (20 million cells/mL fat) added to fat graft (2000× physiological concentration)	Non-enriched standard fat graft (placebo syringe)	50 mL fat harvested for ADSC isolation; SVF isolated by collagenase & cultured 14 days in GMP lab. On the day of grafting: ~8.58×10⁸ cells obtained. Grafts: 30 mL ASC-enriched fat vs 30 mL standard, each injected into opposite upper arms. Bolus subcutaneous injection. MRI used at baseline, post-op, and day 121. Histology done upon surgical removal at day 121	Residual volume at day 121: ASC-enriched: 80.9% (76.6–85.2%) vs Control: 16.3% (11.1–21.4%), Absolute difference: +64.6% (p<0.0001)	Histology: ↑ Adipose tissue (84.3% vs 67.0%), ↑ newly formed connective tissue (5.3% vs 0.5%), ↓ Necrosis (4.6% vs 16.1%), no difference in vessel density. Clinical: uniform graft stability, predictable outcomes	121 days (4 months)	No serious adverse events. Transient redness/swelling from the ASC graft side only in the first 24 h. One subject was excluded due to a misplaced graft	ASC-enrichment produced dramatically higher and more predictable graft survival (>80%). Safe, feasible, and supports stem-cell–enhanced lipofilling as a reliable reconstructive alternative
Kølle et al. [8]	Denmark - Stemform & Aleris Hamlet Hospitals	Randomized, controlled, assessor-blinded clinical trial	N=12 completed (6 ASC-enriched, 6 control). Healthy females; Age median 29 vs 32.5 yrs; BMI median 19.2 vs 20.8 kg/m²; nonsmokers	Healthy adult women seeking primary cosmetic breast augmentation	Ex vivo-expanded ADSCs added to fat grafts ≥20×10⁶ viable ASCs/mL fat	Standard non-enriched fat grafts	Harvesting: initial liposuction 2 weeks earlier (100 mL for ASC culture). Expansion: 10-12 population doublings; viability >90%. Main liposuction: abdomen/thighs/hips. Injection: ≥20×10⁶ ASCs/mL mixed with fat; 14G cannula, multiple planes. Median injected: 222.5 mL/breast (ASC) vs 260 mL (control)	Volume retention at 4 months MRI: ASC 80.2% (66.1–124.2%) vs Control 45.1% (36.5-50.7%) p=.0022	Breast enlargement ratio 2.60-fold ASC vs 1.57-fold control; independent surgeon panel rated ASC superior; predictable contour & symmetry	4 months MRI + 18 months photos (5-year safety planned)	No serious complications; expected swelling/bruising only. No infections, no oil cysts on MRI	ASC-enriched fat grafting improves graft retention and produces clinically superior augmentation. Safe and feasible
Rigotti et al. [9]	Brazil & Italy - Federal University of Rio de Janeiro + University of Verona	Prospective, non-randomized, split-face clinical comparative trial	N=13 Age 45-65 yrs (mean 56.2) Sex 11 female, 2 male; nonsmokers	Patients undergoing facelift surgery with aged facial skin requiring rejuvenation	Two ADSC-based interventions: 1) Expanded ADSCs (2×10⁶ cells in 0.4 mL saline) injected subdermally 2) SVF-enriched fat graft (centrifugation-derived SVF pellet mixed with decanted fat)	PRP-enriched fat graft (1:1 fat + PRP)	Harvesting: 30 cc abdominal fat via 3 mm cannula. SVF: centrifuged at 3000 rpm ×3 min → pellet mechanically dissociated. ADSC expansion: one passage, 4-5 weeks; phenotype confirmed. PRP: two-step centrifugation; platelet concentration 5.4-7.3×; calcium/thrombin-activated. Injection: preauricular areas, 1 cm², subdermal. Biopsies: before & 3 months	Dermal regeneration (histology): Expanded ADSCs → strong improvement in collagen & elastic fibers; SVF-enriched fat → similar regenerative effects; PRP → more inflammation, higher vascular reactivity, less regenerative benefit	PRP caused mononuclear infiltrates, edema, vascular basement membrane duplication, nerve reactivity; Expanded ADSCs & SVF improved dermal elastosis, collagen fiber reorganization, papillary dermis elastic network	3 months	No clinical complications, infections, systemic issues	PRP did not enhance regeneration better than ADSCs or SVF. ADSC-expanded and SVF-enriched fat produced clear regenerative improvements
Vester-Glowinski et al. [10]	Denmark - Copenhagen University Hospital Rigshospitalet	Randomized, double-blind, placebo-controlled RCT	N=10 (all completed) Age median 33 yrs (IQR 30-40) Sex 100% female BMI median 24.3 (IQR 22.9-25.7)	Cosmetic breast augmentation with lipofilling	Ex vivo-expanded ADSCs added to fat graft (10 × 10⁶ ASCs/mL) after 17 days of GMP expansion.	Placebo-enriched fat grafting (standard centrifuged fat) to the contralateral breast	Harvesting round 1: abdomen liposuction to isolate SVF for ASC culture (17-day expansion). ASC expansion: 4.4 × 10⁹ viable ASCs; viability ~96.8%, phenotype CD73+, CD90+, CD105+, CD31-, CD45-. Harvesting round 2: water-jet assisted liposuction of hips/thighs for fat graft. Fat processing: centrifuged 100g × 3 min. Injection: 300–350 mL per breast, structural grafting, blinded allocation, 14G cannula	12-month MRI: ASC-enriched 54.0% vs Placebo/control 55.9%, p=0.566 → no difference	4-month retention: 54.3% ASC vs 56.2% control (p=0.552). Imaging: BI-RADS increase in 90% of breasts; benign cysts are equal. Aesthetic: no difference in contour or asymmetry	4 months + 12 months	No serious adverse events. Benign oily cysts in 9/10 patients; no infections, no malignancy; 1 FNAC for benign finding	ASC enrichment did not improve fat graft volume retention. Fat grafting alone achieved ~55% retention. More research needed
Gentile et al. [11]	Italy - University of Rome Tor Vergata	Prospective comparative clinical study (3-arm)	N=33; SVF+fat graft: n=10, age 19-60; PRP+fat graft: n=13, age 19-60; Control (centrifuged fat): n=10, age 21–65. All female	Breast soft tissue defects: unilateral hypoplasia, post-mastectomy reconstruction, post-implant removal	Enhanced SVF fat grafting (e-SVF via Celution system) mixed with washed fat.	Centrifuged fat grafting (Coleman procedure)	Harvesting: 250–1080 mL lipoaspirate via 3 mm cannula. SVF isolation: Celution 800/CRS; yield 4-5 mL SVF; viability >98%. Mixing: SVF added to washed fat (~430 mL total). PRP group: 0.5 mL PRP mixed with 1 mL centrifuged fat	1-year volume retention: SVF-enhanced 63%, PRP-enriched 69%, control 39%	MRI & ultrasound confirmed increased adipose layer thickness; high patient satisfaction; improved 3D projection; natural contour & softness	12 months (MRI + photo assessment)	Minor events only: 1 small cyst & microcalcification; no infections	SVF-enhanced and PRP-supplemented fat grafting significantly improves 1-year breast volume retention vs standard fat grafting; safe with good aesthetic outcomes
Toyserkani et al. [12]	Denmark - Odense University Hospital	First-in-human, open-label, prospective pilot feasibility and safety clinical study	N=10; Age 34–68 yrs (mean 54.5 ± 12.3); Sex: all female; BMI median ~26; Lymphedema duration median 28.5 months	Breast cancer-related lymphedema (BCRL) of he upper extremity (ISL stage I–II) following surgery + radiotherapy	ADRC-assisted fat grafting: freshly isolated adipose-derived regenerative cells via Celution system + subcutaneous fat grafting for scar release	None	Harvesting: ~300 mL lipoaspirate from thighs/abdomen using water-jet assisted liposuction. Processing: ADRC isolation via Celution 800/IV; yield: 5.37×10⁷ ±1.08×10⁷ cells; viability ~83% ±3%. Injection: 20–30 mL fat graft into axillary scar area + ADRCs (5 mL LR, 8 standardized points)	Arm volume: No significant long-term reduction	Symptoms improved: heaviness & tension ↓, DASH score ↓, QoL improved, 50% reduced conservative therapy	6 months	Minor transient AEs: bruising, mild donor-site pain, pruritus, temporary sensory loss; all resolved. No infections, no cancer recurrence	ADRC + fat grafting is safe, feasible, and improves symptoms. Minimal volume reduction, but symptom improvement suggests therapeutic potential. RCTs needed

Interventions involved fresh SVF, ex vivo-expanded ADSCs, ADRCs, or combinations with PRP. Comparator groups included conventional fat grafting, placebo injections, PRP-enriched grafts, or standard medical therapy. Techniques varied widely in fat harvesting, SVF processing, cell expansion protocols, and injection strategies, reflecting adaptation to specific anatomical and clinical contexts.

Primary outcomes focused on fat graft volume retention measured with MRI, CT, or 3D imaging. Secondary endpoints included changes in skin quality, functional outcomes, symptom improvement, and histologic changes. Follow-up ranged from three months to four years.

Quality Assessment

Randomized trials generally demonstrated low risk of bias across key domains in RoB 2. Studies by Yin et al., Kølle et al., Wang et al., and Vester-Glowinski et al. [1,4,7,10] showed strong randomization procedures and objective imaging-based assessments. Some concerns were noted regarding selective reporting or minor deviations from intended interventions. The study by Iglesias et al. [2] showed a high risk of bias due to unblinded assessments and missing data (Table 2).

**Table 2:** Summary of the quality appraisal of the included studies Risk of bias assessments are presented according to the Cochrane RoB 2 tool [15] for randomized controlled trials (RCTs) and ROBINS-I [16] for non-randomized studies. Domains assessed include randomization/confounding, deviations from intended interventions, missing outcome data, outcome measurement, selection/reporting, and classification/other biases. Overall risk of bias is categorized as low risk, some concerns, high risk (RoB 2), or serious risk (ROBINS-I). RCT - randomized controlled trial; ADRC - adipose-derived regenerative cells; CAL - cell-assisted lipotransfer; SVF - stromal vascular fraction; PRP - platelet-rich plasma

Study (author)	Tool	Randomization / confounding	Deviations / interventions	Missing outcome data	Measurement of outcomes	Selection / reporting	Classification / other bias	Overall risk of bias
Wang et al. [1]	RoB 2 (RCT)	Low Risk	Low Risk	Low Risk	Low Risk	Some Concerns	—	Low Risk
Iglesias et al. [2]	RoB 2 (RCT)	Some Concerns	High Risk	Some Concerns	High Risk	Some Concerns	—	High Risk
Jørgensen et al. [3]	ROBINS-I (Non-RCT)	Serious	Moderate	Low	Moderate	Serious	Moderate	Serious Risk
Yin et al. [4]	RoB 2 (RCT)	Low Risk	Low Risk	Low Risk	Low Risk	Some Concerns	—	Low Risk
Yoshimura et al. [5]	ROBINS-I (Non-RCT)	Serious	Low	Low	Low	Moderate	Moderate	Serious Risk
Yoshimura et al. [6]	ROBINS-I (Non-RCT)	Serious	Low	Low	Low	Moderate	Serious	Serious Risk
Kølle et al. [7]	RoB 2 (RCT)	Low Risk	Low Risk	Low Risk	Low Risk	Low Risk	—	Low Risk
Kølle et al. [8]	RoB 2 (RCT)	Low Risk	Some Concerns	Low Risk	Low Risk	Low Risk	—	Low Risk
Rigotti et al. [9]	ROBINS-I (Non-RCT)	Serious	Low	Low	Low	Serious	Serious	Serious Risk
Vester-Glowinski et al. [10]	RoB 2 (RCT)	Low Risk	Low Risk	Low Risk	Low Risk	Low Risk	—	Low Risk
Gentile et al. [11]	ROBINS-I (Non-RCT)	Serious	Moderate	Low	Moderate	Serious	Serious	Serious Risk
Toyserkani et al. [12]	ROBINS-I (Non-RCT)	Serious	Moderate	Low	Moderate	Serious	Moderate	Serious Risk

All nonrandomized studies were judged to have a serious risk of bias under ROBINS-I. Common limitations included confounding, lack of control groups, heterogeneity in patient characteristics, and reliance on subjective clinical or photographic assessments. Reporting bias was frequently observed.

Fat Volume Retention and Graft Survival

Most randomized studies showed higher fat retention with ADSC or SVF enrichment. Kølle et al. [7] reported 80.9% retention with ADSC enrichment compared with 16.3% in controls, and their subsequent trial showed similarly superior retention in ADSC-enriched breast grafts [8]. Yin et al. [4] observed greater volume differences with SVF enrichment at six months, while Wang et al. [1] found both ADSC- and SVF-enriched grafts outperformed conventional grafting. Vester-Glowinski et al. [10], however, reported no significant difference at 12 months between ADSC-enriched and placebo-enriched fat.

Nonrandomized studies reported similar trends. Gentile et al. [11] found 63% retention with SVF-enriched fat and 69% with PRP-enriched fat compared with 39% with standard grafting. Yoshimura et al. [5] reported reduced late resorption and a more stable contour in CAL patients.

Skin Quality and Dermal Regeneration

SVF-enriched grafts improved skin texture and wrinkle severity, as demonstrated by objective imaging in Yin et al. [4]. Rigotti et al. [9] showed enhanced collagen organization and elastic fiber architecture after ADSC or SVF enrichment, while PRP produced vascular changes and inflammatory infiltration without comparable regenerative benefit. Yoshimura et al. [5] reported improved skin texture and prolonged soft-tissue correction in CAL-treated facial lipoatrophy.

Functional Outcomes in Reconstructive Indications

In localized scleroderma, ADSC-enriched grafts provided superior fat retention and aesthetic improvement compared with SVF-assisted and conventional grafting [1]. In systemic sclerosis, Iglesias et al. [2] showed reductions in digital ulcers and pain after SVF treatment.

Two trials evaluating ADRC-assisted grafting for breast cancer-related lymphedema demonstrated improvements in symptoms such as heaviness, tension, and disability scores, with effects sustained up to four years [3,12]. Neither study showed significant reductions in objective limb volume.

Patient Satisfaction and Aesthetic Outcomes

High satisfaction rates were reported across aesthetic and reconstructive applications. Independent surgeon evaluations favored ADSC-enriched breast augmentation results [8]. Cosmetic improvement and patient satisfaction were consistently high in facial contouring, breast augmentation, and CAL-treated lipoatrophy patients [4,6,11].

Safety Outcomes

Across all studies, serious adverse events attributable to ADSC, SVF, or ADRC enrichment were not reported. Minor complications such as bruising and swelling were transient [3,12]. No increases in infection, seroma, oil cysts, or oncologic recurrence were observed [1,4,7,10]. Occasional benign cysts, microcalcifications, or isolated fat necrosis were reported with no enrichment-related pattern [5,6].

Histologic and Mechanistic Findings

Histologic analyses consistently showed improved graft quality with ADSC or SVF enrichment. Kølle et al. [7] found greater adipocyte viability and less necrosis in enriched grafts. Rigotti et al. [9] demonstrated enhanced dermal collagen and elastic fiber organization after ADSC or SVF enrichment. Gentile et al. [11] reported increased cell proliferation and adipogenic signaling with PRP.

## Conclusions

This systematic review shows that ADSC-, SVF-, and ADRC-enhanced fat grafting consistently improves graft retention, tissue quality, and patient-reported outcomes across aesthetic and reconstructive applications. These advantages align with emerging evidence supporting the regenerative functions of adipose-derived cells, including their roles in vascular support, immune modulation, and extracellular matrix repair. Safety profiles were favorable, with no indication of increased complications. However, differences in study designs, cell processing methods, and regulatory barriers continue to limit standardization and broader implementation. Ongoing high-quality research that integrates mechanistic understanding with robust clinical evaluation is needed to refine these techniques and ensure durable, reproducible results in clinical practice.
